# Familial Aggregation of Urinary Tract and Bone Tumors: Searching for a Syndrome

**DOI:** 10.1155/2012/107894

**Published:** 2012-05-20

**Authors:** Andreas Frings, Jochen B. Geigl, Bernadette Liegl-Atzwanger, Andreas Leithner

**Affiliations:** ^1^Department of Orthopedic Surgery, Medical University of Graz, Auenbruggerplatz 5, 8036 Graz, Austria; ^2^Institute of Human Genetics, Medical University of Graz, Harrachgasse 21/8, 8010 Graz, Austria; ^3^Institute of Pathology, Medical University of Graz, Auenbruggerplatz 25, 8036 Graz, Austria

## Abstract

Positive family anamnesis is an important risk factor for cancer, and therefore further investigations need to be done if familial aggregation of cancer is observed. Due to a rare combination of urinary tract and bone tumors occurring in the family presented herein we hypothesized a hereditary predisposition and thus, Li-Fraumeni syndrome was considered to be the most likely differential diagnosis. To confirm Li-Fraumeni syndrome, we set out to investigate this case by analyzing the tumor suppressor gene *p53*. However, taking into account all the diagnostic results obtained, Li-Fraumeni syndrome could not be confirmed, but there is still uncertainty regarding a definitive diagnosis.

## 1. Introduction

Positive family anamnesis is an important risk factor for cancer, and therefore further investigations need to be done if a familial aggregation of cancer is observed. Due to a rare combination of primary tumors occurring in the family presented herein [1], we assumed a hereditary predisposition and thus, Li-Fraumeni syndrome (LFS) was considered to be the most likely differential diagnosis. First reported by Li and Fraumeni in 1969 [2], this syndrome is characterized by its onset at an early age and an autosomal dominant mode of inheritance with germline mutations at the tumor suppressor gene *p53* [3].

To confirm the hypothesized diagnosis of LFS, we set out to investigate this case by analyzing the tumor suppressor gene *p53*.

## 2. Case Presentation

**Figure 1:**
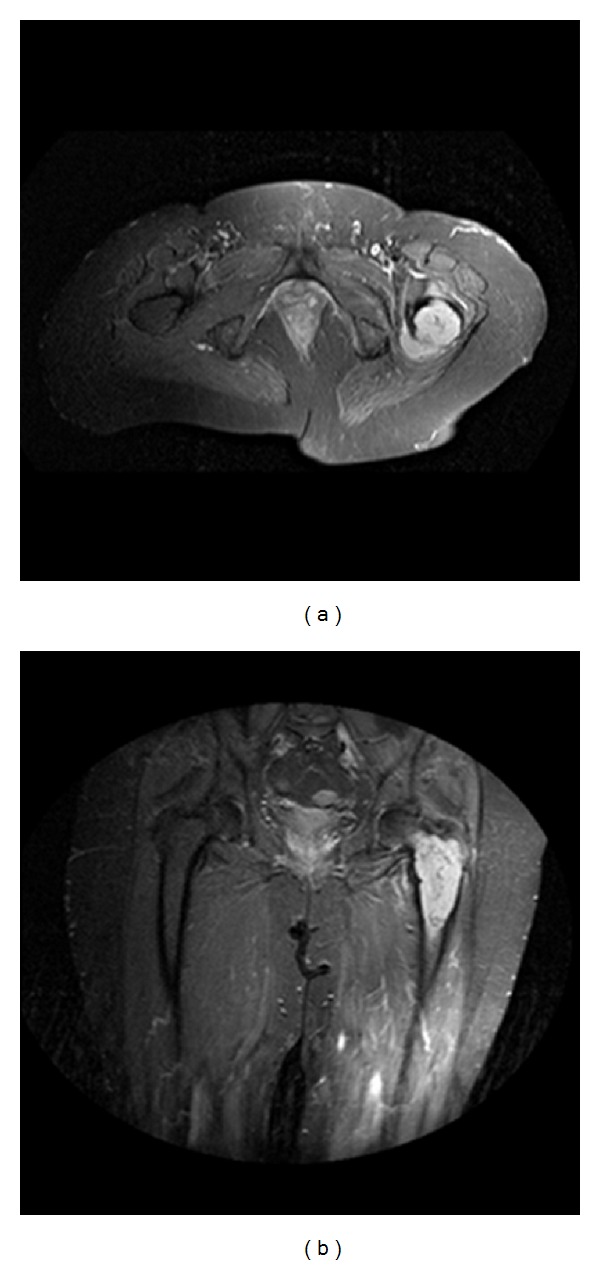
Preoperative MRI showing the left femur of a 76-year-old Caucasian female with diagnosis dedifferentiated chondrosarcoma (a) anterior-posterior axial view, (b) anterior-posterior front view.

A 76-year-old Caucasian female was referred to our department due to a 6-month history of constant aching pain over her left hip region. Imaging features were very typical of a primary bone tumor and thus, as tentative diagnosis chondrosarcoma was made by the reporting radiologist (Figure 1). Subsequently performed tumor staging revealed the tumor as being a primary with an otherwise clear bone scan, CT chest, and abdomen. There was no evidence of metastatic disease. However, imaging of the kidneys suggested possible renal cell carcinoma involving the right kidney.

**Figure 2:**
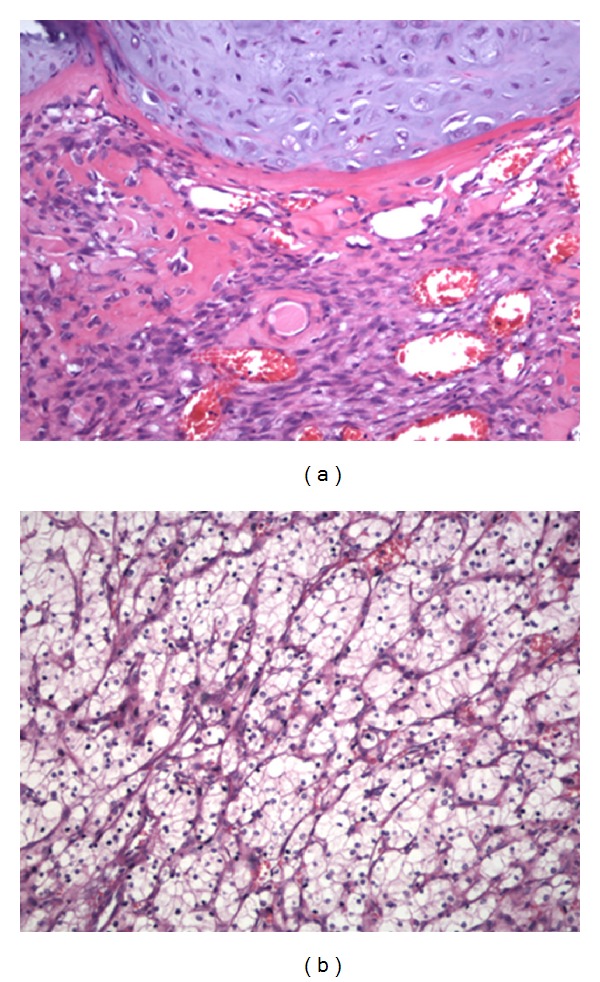
(a) Biopsy specimen of the proximal left femur of a 76-year-old Caucasian female with diagnosis dedifferentiated chondrosarcoma (400x), H.E. (b) same patient, clear-cell renal cell carcinoma of the right kidney (400x), H.E.

Biopsy specimen of the proximal left femur revealed a biphasic tumor composed of a cartilaginous component with abrupt transition into a noncartilaginous malignant mesenchymal component (Figure 2). The diagnosis of a dedifferentiated chondrosarcoma was made. The dedifferentiated part was consistent with an osteosarcoma.

The patient underwent limb salvage surgery, performed according to standardized local protocol by surgeons specialized in oncologic surgery, followed by endoprosthetic replacement. Wide surgical margins were achieved. Although the patient was reasonably fit, adjuvant chemotherapy was not given due to the patient's age.

During the further course of treatment, the suspicious condition in the right kidney, that had already been present during initial tumor staging prior to limb salvage surgery, was diagnosed as clear-cell renal cell carcinoma (Figure 2) and immediately subjected to nephrectomy. There was no evidence of metastatic disease. After more than 5 years of followup the patient is still free of disease.

**Figure 3:**
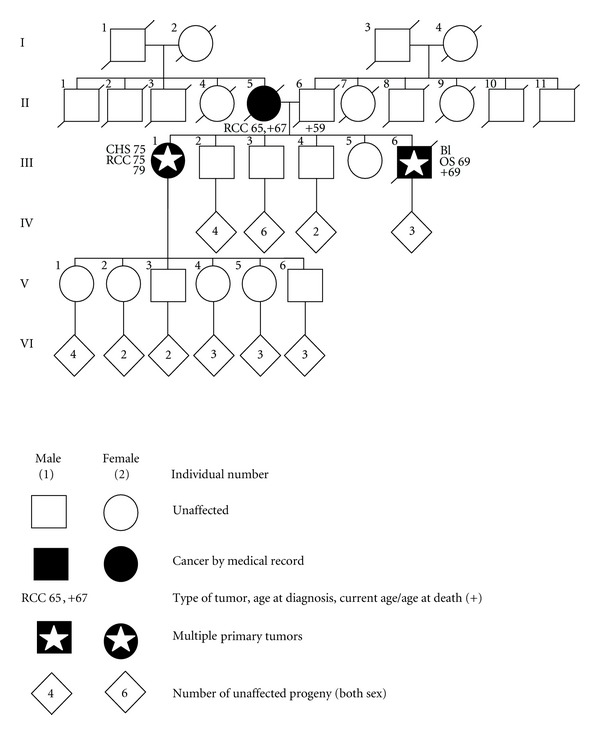
Pedigree of the family presented, RCC: renal cell carcinoma, CHS: chondrosarcoma, OS: osteosarcoma, BL: urothelial carcinoma of the bladder.

**Figure 4:**
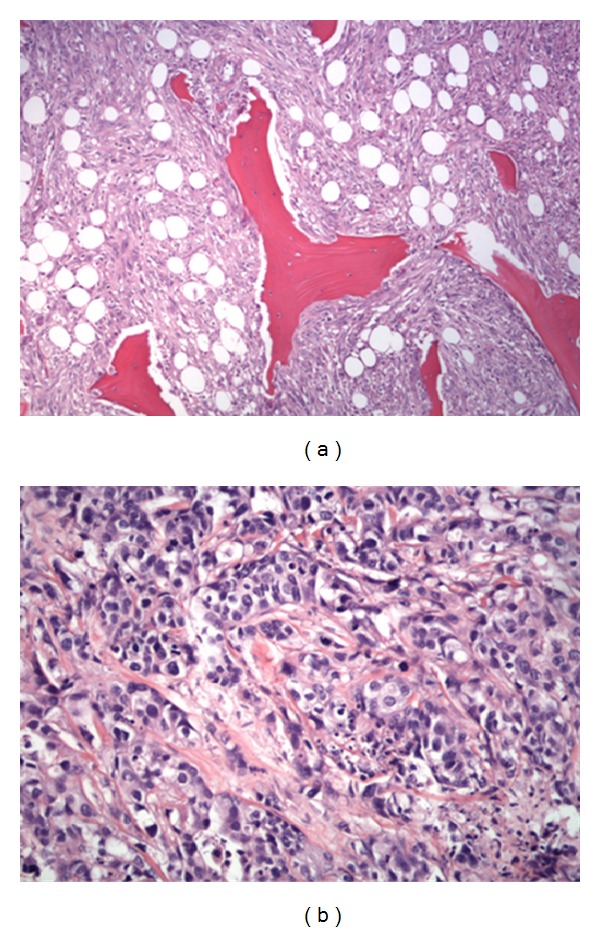
(a) Biopsy specimen of the proximal left femur of a 69-year-old Caucasian male with diagnosis fibroblastic osteosarcoma (200x), H.E. (b) same patient, invasive medium- to-low differentiated urothelial carcinoma of the bladder (400x), H.E.

Besides the current complaints described above, the patient underwent hysterectomy and cholecystectomy years ago. She could not remember the reason and there is no documentation discussing why those operations were performed. However, her medical family history (Figure 3) revealed that her youngest brother started suffering from invasive medium- to-low differentiated urothelial carcinoma (Figure 4) of the bladder when he was 67-year-old. He died at age 69 of a fibroblastic osteosarcoma (Figure 4) located in his left femur. Their mother presented with renal cell carcinoma as well when she was 65-year-old (specimen not available).

To assess the hypothesized diagnosis of LFS, DNA was isolated from blood samples of the female index patient, and we analyzed the tumor suppressor gene *p53* using semiquantitative, multiplex ligation-dependent probe amplification (MLPA, MRC-Holland) to rule out alterations of the *TP53* gene. Moreover, the entire coding (exons 2–11) region and flanking intron regions of *TP53* were sequenced. Since both MLPA and sequenciation did not reveal pathologic changes of the *TP53* gene, the GAG-banding pattern derived from cell division was analyzed as well. However, chromosomal aberrations were not detected in the cytogenetic analysis neither for structural or numerical reasons. Taking into account all the results obtained, the diagnosis of LFS could not be confirmed, but there is still uncertainty regarding a definitive diagnosis in terms of a possible new genetic syndrome.

## 3. Discussion

Due to a rare combination of urinary tract and bone tumors occurring in the family presented herein, we hypothesized a hereditary predisposition and thus, the tentative diagnosis Li-Fraumeni syndrome was made. 

In fact, positive family anamnesis is an important risk factor for cancer. The genetic defect in LFS is a germline mutation at the tumor suppressor gene *p53* with an autosomal dominant mode of inheritance. Accordingly, children of affected parents are at 50% risk of being mutation carriers. As the family pedigree shown in Figure 3 points out that there are a lot of children, a diagnosed syndrome could be very helpful to reduce risk for unaffected offspring. However, none of the offspring has yet developed cancer (Figure 3).

Due to a variety of neoplasms and the family history reported herein, LFS was strongly considered nevertheless. In contrast to other syndromes, for instance neurofibromatosis, tuberous sclerosis, or von Hippel-Lindau syndrome, LFS is characterized by various but characteristic multiple primary tumors [1]. Accordingly, significant differences in the distribution of bone tumors, soft tissue sarcomas, breast cancer, leukemia, melanoma, lung cancer, tumors of the gastrointestinal tract and the pancreas should be expected compared with the general population. Although an early age at onset is frequent, patients are also at increased risk of developing multiple primary late-onset malignancies [4]. The overall lifetime risk of cancer is estimated close to 80% in patients with pathologic changes of the *TP53* gene; approximately 40% develops cancer during the first two decades of life [5, 6].

Further explanations like variable expressivity and germline mosaicism or de novo mutation in the patient brother's *TP53* gene were considered but finally denied due to low probability. Nonmedical explanations, including alternate paternity or maternity or undisclosed adoption, were explored and ruled out by means of family examination. Furthermore, a nonsyndromic cause of familial cancer is still being reasonable as pathologic changes of the *TP53* gene have not been proven. Based on current data, some minor changes found in the DNA sequence can be classified as polymorphism without pathologic significance; however, current data may be subject to change. A genetic predisposition, perhaps due to other, currently unknown factors may still not be excluded. Besides, MLPA and sequenciation as methods for genetic testing may perhaps be too limited. However, these tools are standard procedures in our institutes.

As the case reported herein implies that families with highly suspicious medical family history should be closely monitored to minimize disease-related morbidity. Clinicians should therefore be aware of genetic syndromes as differential diagnosis. Further, similar, familial cases should be reported to address the question of a possible new genetic syndrome.
